# Development of an Antibody to Bovine IL-2 Reveals Multifunctional CD4 T_EM_ Cells in Cattle Naturally Infected with Bovine Tuberculosis

**DOI:** 10.1371/journal.pone.0029194

**Published:** 2011-12-22

**Authors:** Adam O. Whelan, Bernardo Villarreal-Ramos, H. Martin Vordermeier, Philip J. Hogarth

**Affiliations:** TB Research Group, Animal Health and Veterinary Laboratories Agency, Addlestone, Surrey, United Kingdom; Institute of Infectious Diseases and Molecular Medicine, South Africa

## Abstract

Gaining a better understanding of the T cell mechanisms underlying natural immunity to bovine tuberculosis would help to identify immune correlates of disease progression and facilitate the rational design of improved vaccine and diagnostic strategies. CD4 T cells play an established central role in immunity to TB, and recent interest has focussed on the potential role of multifunctional CD4 T cells expressing IFN-γ, IL-2 and TNF-α. Until now, it has not been possible to assess the contribution of these multifunctional CD4 T cells in cattle due to the lack of reagents to detect bovine IL-2 (bIL-2). Using recombinant phage display technology, we have identified an antibody that recognises biologically active bIL-2. Using this antibody, we have developed a polychromatic flow cytometric staining panel that has allowed the investigation of multifunctional CD4 T-cells responses in cattle naturally infected with *M. bovis*. Assessment of the frequency of antigen specific CD4 T cell subsets reveals a dominant IFN-γ^+^IL-2^+^TNF-α^+^ and IFN-γ^+^ TNF-α^+^ response in naturally infected cattle. These multifunctional CD4 T cells express a CD44^hi^CD45RO^+^CD62L^lo^ T-effector memory (T_EM_) phenotype and display higher cytokine median fluorescence intensities than single cytokine producers, consistent with an enhanced ‘quality of response’ as reported for multifunctional cells in human and murine systems. Through our development of these novel immunological bovine tools, we provide the first description of multifunctional T_EM_ cells in cattle. Application of these tools will improve our understanding of protective immunity in bovine TB and allow more direct comparisons of the complex T cell mediated immune responses between murine models, human clinical studies and bovine TB models in the future.

## Introduction

Tuberculosis (TB) caused by infection with *Mycobacterium tuberculosis* or *Mycobacterium bovis* remains one of the most important infectious diseases of man and animals respectively, and continues to inflict a huge cost in humans and animals in both health and financial terms [1]. In the United Kingdom (UK), bovine TB continues to be a significant and growing problem despite the long term use of a test and slaughter control policy based primarily on the tuberculin skin test [2]. Consequently, the British government has acknowledged and supported development of cattle vaccines, and associated improved diagnostic reagents, as research priorities [3].

Gaining a better understanding of the T cell mechanisms underlying natural immunity following infection would help to identify immune correlates of disease progression and facilitate the rational design of improved diagnostic strategies and also have impact on TB vaccine development.

Studies in human and murine models demonstrate that IFN-γ and TNF-α play a central role in protection against mycobacterial disease [4] illustrating the importance of T-cell mediated immune responses in TB. Studies on immune responses to infectious diseases have recently identified and described an important role for multifunctional T cells that co-express IFN-γ, TNF-α and IL-2. For example, multifunctional T cells associate with non-progressors in HIV infection [5], characterise protection in the lungs of influenza infected mice [6] and represent a correlate of protection in a murine leishmania vaccination/challenge model [7]. More recently, multifunctional T cells have also been described in *M. tuberculosis* infection and vaccination models in mice [8], [9], non-human primates (NHP) [10] and human vaccination studies [7], [11]. These studies show a correlation between the frequencies of these cells and the expression of protective immunity. In studies of human infection however, the role of multifunctional T cells remains more cryptic as they may represent a correlation with active disease [12], [13], [14].

To date, it has not been possible to assess the contribution of these multifunctional CD4 T cells in cattle as an important reagent missing from the bovine immunology repertoire has been monoclonal antibodies that recognise biologically active bovine IL-2 (bIL-2). Measurement of bIL-2 has previously only been possible using indirect methods such as IL-2 bioassays [15], [16] or by measurement of mRNA. Furthermore, whilst intracellular cytokine staining (ICS) methods have been developed to characterise bovine T-cells that express IFN-γ [17], [18], phenotypic characterisation of individual T-cells with multifunctional capabilities has not been reported for cattle.

Here we describe the first use of a recombinant human antibody fragment that detects the expression of native bIL-2. We demonstrate its application in a novel multiparametric flow cytometric staining panel that recognises IFN-γ, TNF-α and IL-2 in combination with markers for T cell memory to assess the frequency of TB antigen-specific multifunctional CD4 T cells in TB infected cattle. We report the identification of multifunctional CD4 T cells in cattle. These cells produced IFN-γ, IL-2 and TNF-α and exhibited a characteristic CD44^hi^ CD62L^lo^ CD45RO^+^ T effector memory (T_EM_) phenotype.

## Results

### Generation of recombinant monoclonal antibodies recognising native bIL-2

Recombinant monoclonal antibodies with specificity towards bIL-2 were generated using the propriety HuCal phage display technology (AbD Serotec). Initially, full length bIL-2 was expressed and purified from *E. coli* and this product was used to screen the HuCal antibody libraries. Using this approach, no evidence for antigen or pokeweed mitogen induced bIL-2 responses were detected by any of these antibody clones (data not shown).

**Figure 1:**
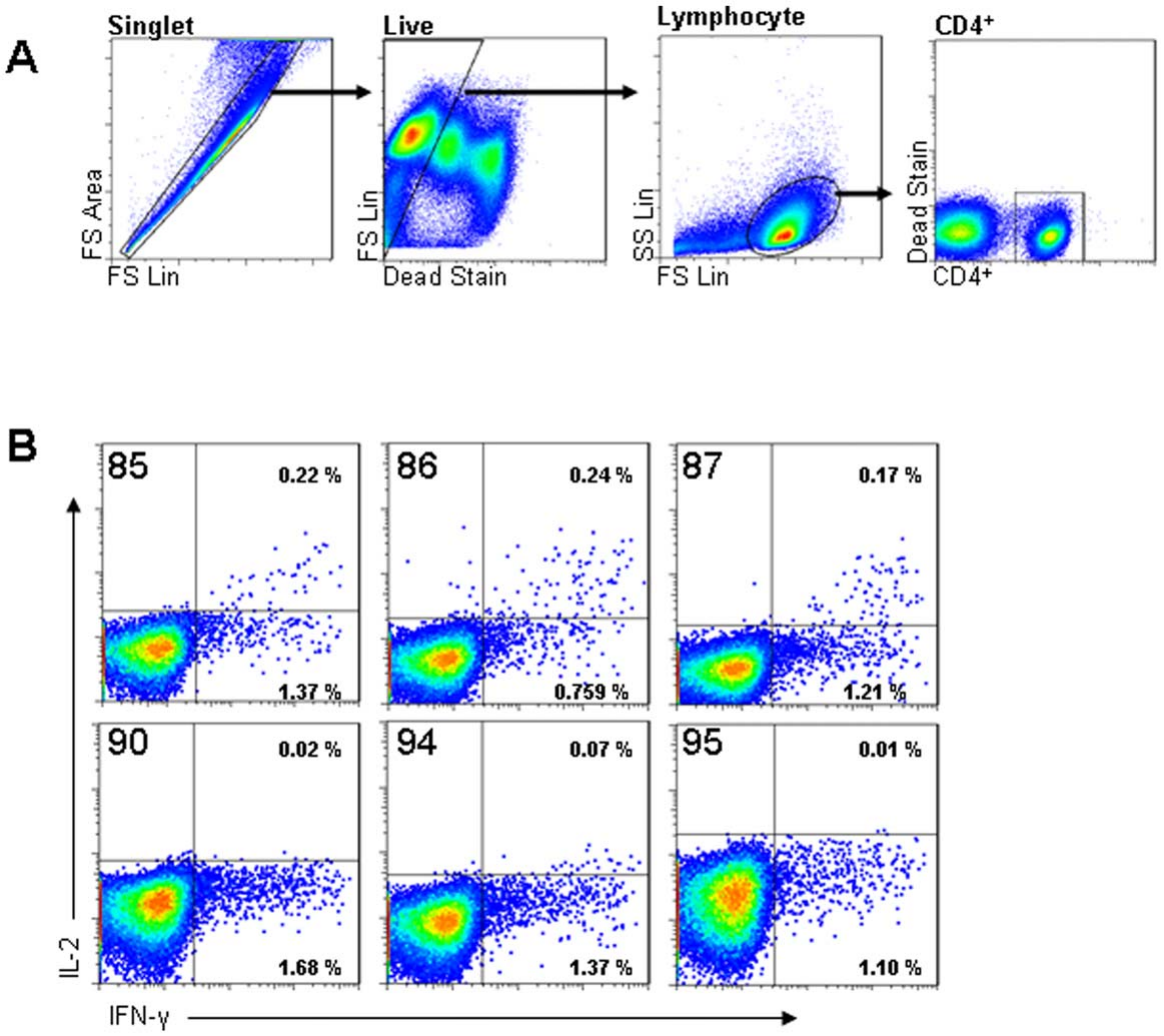
Identification of recombinant antibodies with specificity for bIL-2. PBMC from a cow naturally infected with *M. bovis* were cultured in the presence of PPD-B to allow screening of candidate bIL-2 by ICS flow cytometry. Panel A shows the histogram gating strategy used to interrogate responses in singlet, live CD4^+^ lymphocytes. Panel B shows the measurement of detectable IL-2 and/or IFN-γ within the CD4^+^ population for each of 6 candidate IL-2 antibody clones. The clone number is shown in the top left corner of each histogram and the percentage of CD4^+^ cell in which co-expression of IFN-γ and IL-2 could be detected is shown in the top right of each histogram. Data are representative of 1 of 2 independent experiments.

A second strategy of antibody selection was then performed using a recombinant bIL-2 expressed and purified from a mammalian culture system. On this occasion, 6 clones demonstrating recognition of this recombinant bIL-2 were provided for further evaluation in cattle. These clones, identified as clone 85, 86, 87, 90, 94 and 95 respectively, were evaluated using antigen stimulated PBMC from a naturally infected cow in which we had previously demonstrated bovine purified protein derivative (PPD-B) specific CD4^+^ IFN-γ ICS responses. CD4^+^ cells were interrogated for their antigen induced expression of IL-2, as detected by each of these clones, in combination with ICS co-staining for IFN-γ. Three of the IL-2 antibody clones, 85, 86 and 87, detected PPD-B induced responses whilst the remaining 3 demonstrated little or no response (Figure 1). The proportion of CD4^+^ cells recognising the bIL-2 antibody in medium-alone stimulated PBMC cultures was ≤0.01% for all clones indicating that responses detected by clones 85, 86 and 87 were antigen specific. These data are representative of one of two independent experiments. Interestingly, for those clones that detected an antigen induced response, the IL-2^+^CD4^+^ cells predominantly co-expressed IFN-γ (Figure 1). Based on these data, clone 86 was selected for optimisation of multifunctional ICS staining assays.

### Antigen specific identification of bovine CD4^+^ PBMC producing IFN-γ, IL-2 and TNF-α

**Figure 2:**
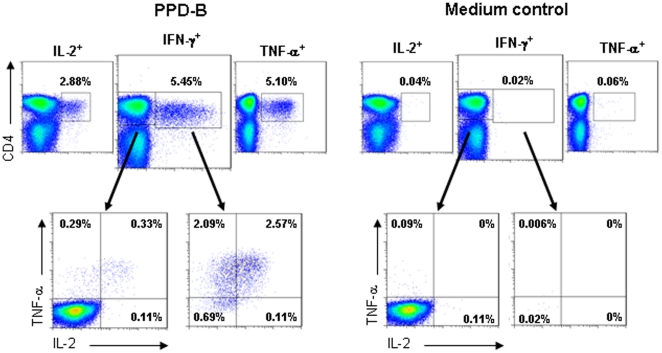
Identification of multifunctional IFN-γ, IL-2 and TNF-α CD4^+^ cells in natural *M. bovis* infection. PBMC from naturally *M. bovis* infected cattle were cultured in the presence of either PPD-B or medium and the co-expression of IFN-γ, IL-2 and TNF-α determined by ICS flow cytometry. Histograms were gated on singlet, live lymphocytes and then all CD4^+^ cells analysed for all combinations of cytokine productivity. The upper histograms show the total proportion of CD4^+^ cells staining for expression of IFN-γ, IL-2 or TNF-α following stimulation with PPD-B (left panel) or medium control (right panel). Subgating of the IFN-γ^+^ and IFN-γ^−^ CD4^+^ cells provides histograms that represent all possible functionalities for the expressing of the 3 cytokines, as shown in the lower histograms. The numbers indicate percentage of CD4^+^ cells and data are representative of 1 of 10 naturally infected cattle.

PBMC were isolated from tuberculin skin-test positive cattle (n = 10) that had been naturally exposed to bovine TB. *M. bovis* infection in these animals was confirmed by the presence of visible TB lesions during subsequent post mortem examination. PBMC from these cattle were stimulated with or without bovine PPD-B and subsequently interrogated by ICS for IL-2, IFN-γ, and TNF-α expression by flow cytometric analysis. The gating strategy and PPD-B induced cytokine expression is shown for one representative animal (Figure 2). In the absence of antigen stimulation, the median percentage of CD4^+^ cells expressing either IFN-γ, IL-2 or TNF-α in these infected animals was 0.04% (range, 0.01–0.09), 0.03% (range, 0.01–0.09) and 0.10% (range, 0.08–0.11) respectively. In contrast, PPD-B induced strong cytokine expression in CD4^+^ cells from all infected cattle such that the median percentage of CD4^+^ cells expressing IFN-γ, IL-2 or TNF-α was 1.84% (range, 0.42–5.58), 1.06% (range, 0.38–3.11) and 2.20% (range, 0.65–5.23) respectively.

To confirm that the PPD-B stimulated responses were detecting antigen specific memory induced by TB infection, the staining panel was also evaluated in non-infected control cattle (n = 5). The median percentage of PPD-B stimulated CD4^+^ cells expressing either IFN-γ, IL-2 or TNF-α was 0.04% (range, 0.02–0.09), 0.03% (range, 0.03–0.09) and 0.10% (range, 0.08–0.11) respectively. These responses were not significantly different from those in the no-antigen control cultures (ρ>0.05, nonparametric paired t-test). PBMC from all control cattle induced detectable cytokine responses to SEB mitogen stimulation (data not shown).

### Analysis of multifunctional T cell subsets in naturally infected cattle reveals a dominant IFN-γ^+^IL-2^+^TNF-α^+^ and IFN-γ^+^ TNF-α^+^ response

**Figure 3:**
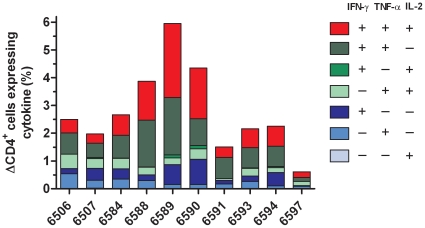
Characterisation of antigen-specific multifunctional CD4^+^ responses in cattle naturally infected with *M. bovis*. PBMC from naturally *M. bovis* infected cattle were cultured in the presence of either PPD-B or medium and the co-expression of IFN-γ, IL-2 and TNF-α determined by ICS flow cytometry. The percentage of PPD-B stimulated CD4^+^ cells expressing all possible combinations of each the 3 cytokines is represented as a stacked bar graph for each of 10 naturally infected cattle (medium control response subtracted).

Using the gating strategy illustrated in Figure 2, it was possible to determine the functionality of antigen specific CD4^+^ cells with respects to their expression of IFN-γ, IL-2 and TNF-α. PPD-B specific cytokine responses for each of the 7 possible subset combinations are shown for each of the infected cattle in Figure 3. The magnitude and proportion of different subset responses were variable between animals, but all displayed a mixture of responses, with multifunctional CD4^+^ IFN-γ^+^IL-2^+^TNF-α^+^ (median 0.76%; range, 0.20–2.66%) and IFN-γ^+^IL-2^−^TNF-α^+^ (median 0.76%; range, 0.14–1.51%) responses predominating (Figure 3). The PPD-B induced response in the confirmed disease free control cattle was less than 0.01% of CD4^+^ T cells for any of the 7 possible subsets (data not shown).

### Multiple cytokine producing cells demonstrate a higher quality of response than single producers

**Figure 4:**
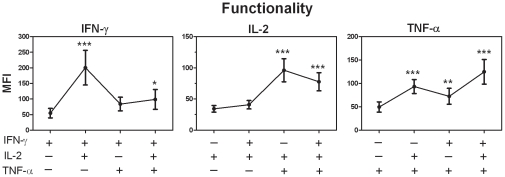
Multifunctional CD4^+^ cells demonstrate a better improved quality of effector cytokine response. PBMC from naturally *M. bovis* infected cattle were cultured in the presence of PPD-B and the expression of IFN-γ, IL-2 and TNF-α determined by ICS flow cytometry. The Median Fluorescence Intensity (MFI) for all single, double and triple cytokine producing CD4^+^ cells were determined. Symbols represent the mean MFI (± SEM) of each of the indicated CD4^+^ functional phenotypes for 10 naturally infected cattle. Significant differences between single and multifunctional phenotypes was determined by Repeat Measures ANOVA with Tukey multicomparison post-analysis test analysis (* ρ<0.05, ** ρ<0.01, *** ρ<0.001).

Median Fluorescence Intensity (MFI) of cells analysed by ICS is a direct correlate of the physical amount of cytokine produced per cell and provides a quantitative assessment of the quality of the cytokine response for different cells. Therefore, we measured IFN-γ, IL-2 and TNF-α MFI for PPD-B stimulated PBMC from naturally infected cattle for each subset of CD4^+^ T cells producing the 7 possible cytokine combinations (Figure 4).

For IFN-γ, the amount of cytokine produced was lowest in single producers (MFI, 55); highest in IFN-γ^+^IL-2^+^ cells (MFI, 200), and intermediate in IFN-γ^+^TNF-α^+^ (MFI, 84) and triple positive cells (MFI, 99). For the IFN-γ^+^IL-2^+^ and triple positive cell populations, this increase in MFI over the single IFN-γ producers was significant (ρ<0.001 and ρ<0.05 respectively). IL-2 showed a highly significant increase in MFI in the IFN-γ^+^TNF-α^+^ and triple producers (ρ<0.001). Finally, all double and triple positive TNF-α producing subsets showed significant higher MFI compared with the single TNF-α producers. Thus, the expression of IFN-γ, IL-2 and TNF-α was significantly greater in the multifunctional (double & triple) populations that in any of the mono-functional populations and there was an overall trend of higher quality of response as the cells increased in function.

### Multifunctional CD4^+^ T cells express a CD44^hi^CD45RO^+^CD62L^lo^ phenotype

**Figure 5:**
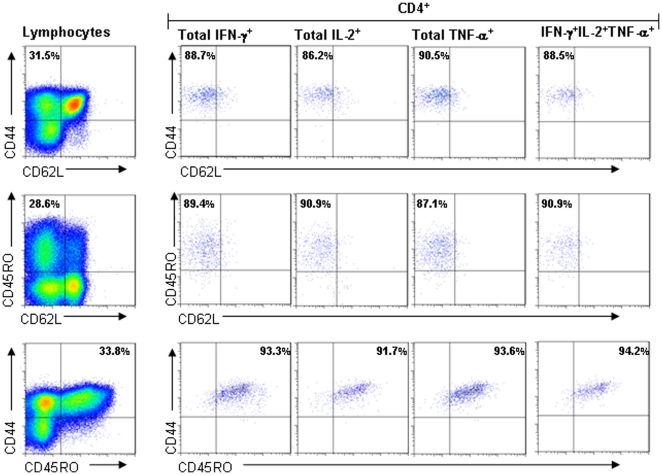
Characterisation of memory markers reveals a characteristic T_EM_ phenotype for multifunctional CD4^+^ cells in naturally infected cattle. PBMC from naturally *M. bovis* infected cattle were stimulated with PPD-B and stained for production of cytokine by ICS flow cytometry. Histograms were first gated on singlet, live lymphocytes and gating strategies applied to allow phenotyping of cells for expression of CD44, CD45RO and CD62L. The quadrant gate defining expression of CD44 and CD62L (top panel), CD45RO and CD62L (middle panel) or CD44 and CD45RO (bottom panel) was determined using the total live-lymphocyte population, as shown for each surface marker combination in the histograms on the far left of the panel. This quadrant gating strategy was used to determine the surface phenotype of total CD4^+^IFN-γ^+^, CD4^+^IL-2^+^, CD4^+^TNF-α^+^ and triple functional CD4^+^ cells, as shown in the other histograms. The percentage of cytokine producing CD44^hi^CD62L^lo^, CD45RO^+^CD62L^lo^ and CD44^hi^CD45RO^+^ is shown for each of these CD4^+^ populations. The data is from one representative animal of 5 analysed.

In order to establish the phenotype of the multifunctional cells measured in naturally infected cattle we developed an ICS staining panel which included the phenotypic memory markers CD62L, CD44 and CD45RO. Using this panel, we performed ICS staining of PPD-B stimulated PBMC cultures for a further 5 naturally infected cattle. To determine the gating strategy to define positive and negative staining for the phenotypic markers, we first set the quadrant gate using the lymphocyte populations. We then used this quadrant gate to interrogate all antigen specific cells producing either IFN-γ, IL-2, TNF-α or co-expressing all 3 cytokines, as shown for one representative animal (Figure 5). Approximately 25–30% of lymphocytes showed a T_EM_ phenotype as characterised by the staining phenotypes CD44^hi^CD62L^lo^ (median 26.9%, range 24.7–35.1) or CD45RO^+^CD62L^lo^ (median 25.9, range 20.7–28-6) whilst the antigen specific cytokine producing populations predominantly expressed these T_EM_ phenotypes (Figure 5). The median proportion of CD4^+^IFN-γ^+^, CD4^+^IL-2^+^, CD4^+^TNF-α^+^ and CD4^+^IFN-γ^+^IL-2^+^TNF-α^+^ cells with a CD44^hi^CD62L^lo^ phenotype was 87.3%, 75.0%, 87.5% and 90.9% respectively. The proportion of these cytokine-producing populations with the CD45RO^+^CD62L^lo^ phenotype were comparable (data not shown). Comparing expression of CD44 with CD45RO revealed that >90% co-express these markers with a median proportion of co-expression in the triple functional cells of 95.9% (range, 94.2–98.5) (Figure 5).

## Discussion

A more detailed understanding of immunological responses in cattle infected with *M. bovis* will be of great value in the development of bovine TB vaccines and improved diagnostic reagents. Given the established central role of CD4^+^ T cells in immunity against TB, and the recent identification and description of a variety of roles for multifunctional T cells expressing IFN-γ, TNF-α and IL-2 in a number of disease and vaccination models [5], [8], [9], [10], [19]; we sought to develop a flow cytometry panel which would allow the identification and assessment of multifunctional T cells in cattle.

An essential reagent required to achieve this was a monoclonal antibody that recognised native bIL-2. The development of this reagent in the current study is an important advance both for the study of TB as well as fundamental immunology in cattle.

It is unclear why the generation of bIL-2 antibodies has previously proved to be so problematic, despite notable successes in the generation of antibodies against other important bovine cytokines such as IFN-γ and TNF-α [20], [21]. Our success in identifying an antibody detecting native bIL-2 demonstrates the utility of the HuCal recombinant human antibody library approach [22] for screening for cattle antibodies. It is interesting that the initial screen of the HuCal library using rbIL-2 expressed from an *E. coli* culture system failed to generate any antibodies that recognised native bIL-2. In contrast, screening with rbIL-2 purified from a mammalian culture system successfully generated antibodies recognising native bIL-2. Whilst the epitope, or epitopes, that these antibodies recognise have yet to be defined, this observation suggests the importance of the confirmation and/or posttranslational modification of the IL-2 protein used in the screening process, a factor that may help explain problems generating antibodies previously.

Since the demonstration that multifunctional CD4^+^ T cells define a correlate of protection against *Leishmania major* in mice [7] there has been much interest in their role in TB. Multifunctional CD4^+^ T cells associate with vaccine induced protection against *M. tuberculosis* in mice [8], [9], [23] and non-human primates (NHP) [10] and vaccine induced responses in humans [11], [12], [24]. However, the study of multifunctional CD4+ T cells in TB affected people provides a more complex picture. Sutherland *et al*., [25] and Caccamo *et al*., [14] demonstrated that active clinical TB is associated with an increase in TB-specific multifunctional cells, whilst other reports associate cells co-expressing IFN-γ and IL-2 with latent or successfully treated disease [26], [27].

In naturally infected cattle, we observed a predominance of multifunctional CD4^+^ T-cells in response to antigen stimulation. Consistent with the findings of others [7], [8], [9], [28] these multifunctional cells also expressed more cytokine than monofunctional cells, i.e. a higher quality if response. The dominant presence of antigen specific multifunctional CD4^+^ cells in the present study in infected cattle suggests that their measurement correlates with active disease, in broad agreement with the findings of Sutherland *et. al.,* [25] and Caccamo *et. al*., [14] in humans. However, it should be noted that although we found TB lesions in the study animals, prior to the post mortem examinations, none of the animals demonstrated any clinical symptoms of TB and therefore it is not possible to state the stage of disease in the animals under study. It should be noted however, that the observed responses in the cattle studied did not correlate with the level of pathology observed which makes it less likely that multifunctionality can be correlated with early infection (data not shown). In the recent assessment of multifunctional responses in humans with latent or active TB, Harari *et. al*., reported that active disease was most strongly associated with single TNF-α^+^ CD4^+^ cells whilst latent disease associated with cell co-expressing IFN-γ and IL-2 [26]. In our study, single TNF-α^+^ producing CD4^+^ cells were a relatively minor population. Therefore, it is unclear whether the multifunctional responses detected in naturally infected cattle provides evidence of progressive TB or whether they are more akin to responses observed in people with latent infection.

Analysis of the memory phenotype of the responding multifunctional CD4^+^ T cells revealed them to display a CD44^hi^CD45RO^+^CD62L^lo^ phenotype, which could indicate a T effector (T_Eff_) or T Effector memory (T_EM_) function. The lack of reagents to CCR7 or markers of terminal differentiation such as CD27 in the bovine system prevents the assignation of these cells definitively based on their surface markers, but the concomitant production of IFN-γ and IL-2 infers memory in addition to effector function, and as suggested by others & in agreement with the model proposed by Seder & colleagues, we propose these cells to be T_EM_ [7], [28], [29], [30].

Previous studies on memory responses in cattle have identified antigen-specific CD45RO^+^ [18], [31], [32], CD44^hi^ and CD62L^lo^ [33] CD4^+^ T cells in cattle, although to date only CD45RO expression has been associated with cytokine effector function. CD44 has been identified as a marker of activation in bovine T cells [33] and is a commonly used marker in murine systems, Bovine CD45RO is considered analogous to the human molecule. Our identification that CD44 and CD45RO staining describes the same multifunctional CD4^+^CD62L^lo^ T cells, allows direct comparison of responses across the murine, bovine and human systems.

In summary, we describe here for the first time the development of a monoclonal antibody against bIL-2 & subsequent polychromatic flow cytometry staining panel to identify multifunctional CD4^+^ T cells in cattle. Cattle naturally infected with bovine TB exhibited predominant IFN-γ^+^IL-2^+^TNF-α^+^ and IFN-γ^+^IL-2^−^TNF-α^+^ CD4^+^ T cells, exhibiting a T_EM_ phenotype. Future application of these tools in bovine TB vaccine efficacy trials, as well as in the study of natural infection, has great potential to improve our understanding of protective immunity in bovine TB and allow more direct comparisons of the complex T cell mediated immune responses between murine models, human clinical studies and bovine TB models in the future.

## Materials and Methods

### Ethics

This study and all procedures were approved by the Animal Health and Veterinary Laboratories Agency (AHVLA) Animal Use Ethics Committee (UK Home Office PCD 70/6905) and performed under appropriate personal and project licences within the conditions of the Animals (Scientific Procedures) Act 1986. All animals were housed in appropriate biological containment facilities at the AHVLA.

### Naturally infected and control cattle

To investigate immune responses in cattle naturally exposed to *M. bovis*, tuberculin skin-test reactor cattle were recruited from UK farms with a confirmed history of bovine TB. Animals were identified during routine surveillance operations where their comparative tuberculin skin-test response [(bovine tuberculin)-(avian tuberculin)] >2 mm. To confirm infection status, animals underwent detailed post mortem examination in accordance with previously described procedures [34]. Non-infected control cattle were obtained from TB-free herds located in non endemic areas.

### Generation of Recombinant bIL-2 Monoclonal Antibodies

Human recombinant monoclonal bivalent Fab antibody fragments were generated using the full length protein (minus secretion leader; aa 20–155) of the bovine IL-2 ORF [35] and HuCAL® phage display technology (AbDSerotec, UK). Briefly, following subcloning and sequence verification, recombinant bIL-2 was expressed either as a His-tagged protein or as a Fc-fusion protein. These recombinant proteins were used to screen the HuCal libraries of bi-valent Fab antibodies. Those clones that provided recognition of the bIL-2 were sequenced and the unique clones expressed, purified and supplied for further evaluation in cattle.

### Cell Isolation and Culture

Peripheral blood mononuclear cells (PBMC) were isolated from heparinized cattle blood by Histopaque-1077 (Sigma, UK) gradient density centrifugation. Purified PBMC were cultured at a concentration of 2×10^6^cell/ml in RPMI1640 (Life Technologies, UK) supplemented with 5% Foetal Bovine Serum (Sigma), non-essential amino acids (Sigma), 5×10^−5^ M 2-mercaptoethanol, 100 U/ml penicillin, and 100 µg/ml streptomycin sulphate (Gibco, UK) in the presence of 1∶100 dilution of Lelystad bovine tuberculin purified protein derivative (PPD-B, Prionics, Switzerland). Control cultures containing either medium alone or mitogen (2 µg/ml staphyloccucus entertoxin-B (SEB, Sigma) or 5 ug/ml pokeweed mitogen (Sigma)) were set up in parallel. PBMC were cultured in the presence of PPD-B, mitogen or medium for 6 hours followed by a further 16 hours in the presence of Brefeldin-A (Sigma).

### Flow Cytometry

Cultured PBMC were washed (300 g/5 mins) and stained using one of 3 different staining panels. All staining was performed at 4°C. To allow screening of the initial candidate bIL-2 antibody clones, PBMC were surfaced stained using CD4:Alexa488 and Violet fixable dead cell stain (Invitrogen, UK) and then fixed for 16 hours at 4°C in Cytofix (BD Biosciences, UK). Fixed cells were permeabilised in BD Biosciences Permeabilistation Buffer and then stained intracellularly with IFN-γ-PE and bIL-2 Fab antibody clones followed by secondary staining with anti-human IgG(Fab)-FITC. The same staining strategy was used for all staining panels. To determine multifunctional responses in a panel of naturally infected cattle, PBMC were surfaced stained with CD4-APC-Alexa750 and Violet fixable dead cell stain and intracellularly stained with IFN-γ-PE, IL-2:DyLight649 and TNF-α:Alexa488. To assess memory phenotype of multifunctional cells, PBMC were surfaced stained with CD4:APC-Alexa750, CD62L:eFluor605, CD45RO:PE-Cy7, CD44:eFlour450 and Yellow fixable dead cell stain (Invitrogen) and intracellularly stained with IFN-γ:PE, bIL2:DyLight649 and TNF-α:Alexa488. ICS stained samples were acquired using a CyAn ADP analyser equipped with 405, 488 and 642 nm lasers (Beckman Coulter, USA). Data analysis was performed using FlowJo v7.6 software (TreeStar, USA). To allow interrogation and reporting of ICS responses during screening of IL-2 clones, assessment of multifunctional responses in infected cattle and memory phenotype analysis, the median number singlet, live lymphocyte events analysed was 182 k, 187 k and 218 k respectively.

### Antibody-fluorochrome conjugates

To facilitate detection of unlabelled bIL-2 clones supplied by AbD-Serotec for evaluation in cattle, secondary staining with a goat anti-human IgG(Fab)-FITC conjugate was used (AbD-Serotec). The bIL-2 antibody clone 86 used for further panel development (AbD14386) was supplied conjugated to DyLight649 (AbD-Serotec). IFN-γ-PE (clone CC302) and TNF-α:Alexa488 (clone CC327) antibody conjugates were supplied by AbD-Serotec. CD4 antibody (clone CC8, AbDSerotec) and CD45RO (clone IL-A116; a kind gift of Dr J. Naessens, ILRI) and were custom conjugated with APC-Alexa-750 and PE-Cy7 respectively by Beckman Coulter Inc. (Marseille, France). CD62L antibody (clone CC32, AbDSerotec) was conjugated in-house with eFluor605 according to the manufacturer's instructions (eBioscience Ltd., UK). A cross reactive anti-murine CD44eFluor450 antibody conjugate was supplied by eBioscience. All antibody conjugates were pre-titrated to determine optimal working concentrations.

### Statistical Analysis

Comparison of ICS responses between PPD-B and medium control cultures in TB-free cattle was performed using a non-parametric Wilcoxon matched-pairs signed-rank test. Differences between the magnitude of cytokine responses in single, double and triple-functional cell population were determined using repeated measures ANOVA with Tukey multicomparison post analysis test. Statistical analysis was performed using InStat software (GraphPad Software Inc., USA).
